# Clinical Behavior and Molecular Insights of Secretory Carcinoma of Salivary Glands, a Single Center Experience

**DOI:** 10.1007/s12070-024-04807-4

**Published:** 2024-06-20

**Authors:** Sara Bassani, Denise Fiorini, Miriam Sara Destefanis, Athena Eliana Arsie, Davide Mulone, Albino Eccher, Matteo Brunelli, Filippo Marani, Daniele Monzani, Gabriele Molteni

**Affiliations:** 1 Otolaryngology-Head and Neck Surgery Department, University of Verona, Verona, Italy; 2 Department of Diagnostics and Public Health, Section of Pathology, University and Hospital Trust of Verona, Verona, Italy; 3 Department of Pathology and Diagnostics, University of Modena and Reggio Emilia, Modena, Italy; 4 Azienda Ospedaliera Universitaria Integrata of Verona, Verona, Italy; 5 Otolaryngology and Audiology Unit, department of Medical and Surgical Sciences, IRCCS Azienda Ospedaliero-Universitaria of Bologna, Alma Mater Studiorum - University of Bologna, Bologna, Italy

**Keywords:** Salivary Gland Tumors, Secretory Carcinoma, NTRK3, Head and neck cancer

## Abstract

Objective: the study aimed to characterize the novel entity referred to as secretory carcinoma of the salivary glands. Methods: we comprehensively evaluated 150 patients afflicted by malignant salivary gland tumors who had been under treatment at the University of Verona. Inclusion criteria primarily focused on the availability of paraffin block materials and adequate follow-up data. Subsequently, we conducted a comprehensive Fluorescent In Situ Hybridization (FISH) analysis, utilizing probes targeting NTRK-3, MALM-2, EWRS-1, HER-2, MDM-2, and NTRK1-2. Results: out of the initial cohort, 37 patients met the eligibility criteria for our study. We identified NTRK3 gene rearrangements in four patients (11%), two of whom had mucoepidermoid carcinoma, and the remaining two had acinic cell carcinoma. Notably, none of these patients had initially received a secretory carcinoma diagnosis. The primary treatment approach for all patients entailed surgical parotid gland resection. The overall survival (OS) for patients with NTRK3 rearrangements amounted to 78 months, with a corresponding progression-free survival (PFS) of 73 months. Conclusion: in summary, our case series suggests that secretory carcinomas exhibit a favorable clinical course and underscores the pivotal importance of distinguishing secretory carcinomas from other histological subtypes.

## Introduction

Salivary glands, which produce and release saliva into the oral cavity, include three pairs of major glands (parotid, submandibular, and sublingual) and numerous minor glands lining the oral cavity. Major and minor salivary gland tumors, characterized by varying histology and biological behavior, pose diagnostic and treatment challenges, constituting approximately 3 to 6% of head and neck tumors [1, 2]. While still uncertain, risk factors for salivary gland cancer include previous radiation, viral infections (EBV, HIV), immunosuppression, and genetic factors [3]. Unlike other head and neck tumors, the link between salivary gland tumors and smoking or alcohol consumption is controversial.

Salivary gland neoplasms are predominantly benign or low grade tumors, with minor glands accounting for only 25% of cases but having a higher malignancy rate. Distinguishing malignant from benign tumors is crucial as only 20% of salivary gland tumors are malignant. Symptoms suggestive of malignancy include pain, rapid growth, and nerve involvement with functional deficits. Fine needle aspiration cytology (FNAC), ultrasound (US), computerized tomography (CT), and magnetic resonance imaging (MRI) are used for diagnosis and staging [4].

FNAC, a widely used tool with relatively high sensitivity and specificity, aids in malignancy determination, patient counseling, and surgical planning. Drawbacks include lower sensitivity than specificity and a relatively high rate of non-diagnostic results [5]. The Milan classification categorizes needle aspirations based on the risk of malignancy (ROM) into seven categories [6].

Salivary gland tumor diagnosis can be challenging due to overlapping characteristics. Recent genetic evaluation methods have improved accuracy, leading to better treatments and prognosis [2]. Most of the salivary gland tumors have specific alterations with distinct roles [7].

In 2010, Skálová et al. [8] introduced a novel form of salivary gland cancer characterized by the existence of the ETV6-NTRK3 fusion gene. Its histopathological presentation closely resembles that of secretory carcinoma in the breast. Given their shared ETV6-NTRK3 fusion gene, it was suggested to name this entity “mammary analogue secretory carcinoma” (MASC). In the updated 2017 classification by WHO, this condition is recognized as secretory carcinoma to standardize terminology across various organ sites [9]. The morphological attributes of secretory carcinoma encompass diverse architectural growth patterns, the presence of intracytoplasmic vacuoles, and an absence of intracytoplasmic zymogen granules set against a background rich in mucinous or hemosiderin-laden histiocytes [10]. Immunohistochemical markers such as S100, vimentin, STAT5a, MUC4, and mammaglobin may offer valuable clues in identifying patients suspected of having secretory carcinoma. Nevertheless, none of these markers alone can definitively confirm the diagnosis. The unmistakable indicator is the presence of the ETV6-NTRK3 fusion gene [11]. The primary entities that are crucial in the differential diagnosis of secretory carcinoma include acinic cell carcinoma, polymorphous adenocarcinoma, adenocarcinoma with unspecified characteristics and mucoepidermoid carcinoma [12]. ETV6-NTRK3 fusion gene is characterized by the specific t(12;15)(p13;q25) chromosomal translocation, serving as a vital diagnostic marker, especially in small biopsies. NTRK3 fusions are unique to secretory carcinomas of the salivary gland.

Despite therapeutic advancements, the prognostic significance of NTRK3 fusions requires further research to understand their impact on tumor behavior and patient outcomes. In recent years, several studies have been published on the histopathological characteristics of secretory carcinoma and its subtypes, but little is known regarding the clinical behavior of these tumors. The aim of this paper is therefore to focus on clinical behavior and outcome of patients with secretory carcinoma.

## Materials and Methods

### Cases Selection

For this study, 37 patients were retrospectively enrolled with a histological diagnosis of malignant salivary gland tumor in the decade 2012–2022 at the Department of Otolaryngology and Head and Neck Surgery and the Department of Pathology and Diagnostics, University and Hospital Trust of Verona.

The inclusion criteria in the study are as follows:

- Patients diagnosed with histologically documented malignant tumors of the salivary glands;
- Histological classification based on the 4th Edition of the salivary glands cancer classification of the World Health Organization (WHO);
- Diagnosis between 2012 and 2021;
- FNAC performed before histology;
- Primary tumors of the parotid, submandibular, sublingual, or minor salivary glands of the head and neck;
- Minimum follow-up of 1 year.

The exclusion criteria are as follows:

- Benign tumors of the salivary glands;
- FNAC not done or not found;
- Metastases from other sites located in the salivary glands.

We analyzed a panel of seven molecular alterations for each case using fluorescence in situ hybridization (FISH): HER2, MALM2, MDM2, NTRK1, NTRK2, NTRK3, EWRS1. All the patients involved gave their consent for the participation in the study. The study protocol was approved by the Azienda Ospedaliera Universitaria Integrata of Verona Ethics Committee (approval number 17798_OSS) and conducted in accordance with the the Declaration of Helsinki.

These cases, according to the 4th WHO classification, were divided into histopathological categories. The study’s primary purpose is to describe the distribution of these anomalies in the tumours under study, evaluate the presence of NTRK3 rearrangement in the patient cohort, and understand if there is a correlation between this molecular anomaly and the behaviour of the disease.

We employed two key measures to assess their survival outcomes: Overall Survival (OS) and Progression-free Survival (PFS). OS is defined as the time elapsed from the date of diagnosis until the patient’s last follow-up examination or the date of death. On the other hand, PFS signifies the duration from the initial disease diagnosis during which a patient remains devoid of any signs or symptoms.

### Histological Preparation/Mounting

To facilitate molecular analysis, histological specimens underwent initial light microscopy examination to identify the tissue withdrawal site for the tissue microarray (TMA) and to collect specimens for optimizing the Fluorescent in situ hybridization (FISH) results.

In the TMA technique, we extracted three microsamples of pathological tissue per patient, embedding them in a paraffin block. These blocks accommodated up to ninety-seven microsamples, including thirteen control tissue samples (in our case, placental tissue) used as reference markers during final fluorescence microscopy evaluation. A schematic representation of the TMA grid was created to track patient allocation within the block.

### Fish

FISH allows detection and visualisation of specific nucleic acid sequences in a cytological or histological specimen.

A FISH probe consists of a fluorochrome-labelled deoxyribonucleic acid (DNA) fragment that binds to its complementary target sequence after denaturation. Then, the sample is washed to remove aspecific or undound fragments and counterstained with 4’ 6-diamidino-2-phenylindole (DAPI), an organic fluorochrome binding to the adenin(A)-thymine(T) sequences of the DNA. The interposition of two filters, namely ZyGreen ec: 503 nm, cm 528 and ZyOrange ec 547 nm, cm 572, makes the DAPI-bound sequences readily detectable at the fluorescence microscopy.

The studied genes were the following ones:

- MAML-2 (Mastermind Like Transcription Coactivator 2) upregulates the Noch signalling pathway. Its translocation gives rise to CRTC1 and CRTC3 fusion proteins involved in the development of mucoepidermoid carcinoma;
- MDM2 (E3 ubiquitin protein ligase) is a proto-oncogene coding for an E3 ubiquitin ligase localized in the nucleus where in induces P53 ubiquitination and degradation by proteasomes. The locus of this gene is amplified in many tumours;
- HER2 (Human Epidermal Growth Factor Receptor 2) the gene Erb 2 codes for a tyrosine-kinase membrane receptor belonging to the group of Erb epidermal growth factors receptors. It is involved in signal transduction. When activated, it induces cell survival and proliferation;
- EWRS1 (Ewing Sarcoma RNA Binding Protein) codes for a multifunctional protein involved in numerous processes such as gene expression, cell signalling and RNA transport. Its translocation leads to the formation of chimeric proteins with a tumorigenic potential for Ewing sarcoma, neuroectodermic tumours, and clear cell carcinoma;
- NTRK1, 2, 3 (tyrosine-kinase neurotrophic receptor) code for tyrosine-kinase receptors involved in cell proliferation and differentiation. The fusion gene ETV6 (12p13.2) - NTRK3 (15q25.3) stems from the fusion between the 5’end of ETV6 and the 3’ end of NTRK3 and is present in the secretory carcinoma.

## Results

We evaluated 150 cases of malignant salivary gland tumors, with only 37 met the inclusion criteria useful for the study. Among these select cases, the distribution was as follows: 13 were identified as mucoepidermoid carcinomas, 4 as carcinomas ex pleomorphic adenoma, 7 as adenoid cystic carcinomas, 5 as acinic cell carcinomas, 2 as myoepithelial carcinomas, 2 as squamous cell carcinomas, 1 as adenocarcinoma, 1 as epithelial-myoepithelial carcinoma, 1 as neuroendocrine carcinoma, and 1 as salivary duct carcinoma.

General characteristics of patients and tumors are collected in Table 1.

**Table 1:** Patients and tumors characteristics

Demographics		
Gender	Male: 19	Female 18
Age at diagnosis	Range: 16–97	Mean: 71.32
Milan Classification	**Number**	**Percentage**
I	3	8.11
II	1	2.70
III	15	40.54
IVa	4	10.81
IVb	2	5.41
V	4	10.81
VI	8	21.62
Clinical staging		**Number**
cT1N0M0		12
cT2N0M0		9
cT2N2bM0		2
cT2N2cM0		1
cT3N0M0		7
cT3N2cM0		2
cT4aN0M0		3
cT4bN3bM1		1
Treatment		**Number**
S		26
S + RT		9
S + Protontherapy		1
RT		1

Surgery (S), Radiotherapy (RT)

As shown in Table 2, in our patient pool, the rearrangement of the NTRK3 gene was found in four patients: two diagnosed with mucoepidermoid carcinoma and two with acinic cell carcinoma. According to various research, the current evidence establishes that chromosomal changes called “break-apart” (B) and associated chimera genes are diagnostic when they reach a percentage varying from 10 to 20% of the nuclei analyzed. A gene is considered “Not break-apart” (NB) when it does not show any rearrangement (less than 5% of nuclei). The indication “Augmented signal not break-apart” (auSNB) refers to a signal that is increased but not sufficient to consider the gene as “Break-apart” (between 5% and 10% of nuclei).

NB and auSNB cases are classified as NB, indicating that the gene does not have chromosomal rearrangements.

The classification “Amplified” (A) indicates that the gene is mutated when there is a high copy of signal per nucleus, while “Not amplified” (NA) indicates the absence of amplification of the gene. For example, in the case of HER2, to be considered an oncogene, the gene must have been replicated numerous times, showing several randomly replicated signals of the gene in each nucleus.

The “Mini Break-apart” (miniB) category refers to a situation where the gene signal is separated, with an apparent break. However, the two portions are not far enough apart to be classified as “Break-apart”.

**Table 2:** Description of mutations

Histotype	Site	Studied genes						
		HER2	MALM2	MDM2	NTRK1	NTRK2	NTRK3	EWRS1
Mucoepidermoid carcinoma	Parotid	NA	NB	NA	auSNB	NB	NB	auSNB
Mucoepidermoid carcinoma	Parotid	NA	NB	NA	NB	NB	NB	NB
Mucoepidermoid carcinoma	Parotid	NA	B	NA	NB	NB	B	NB
Mucoepidermoid carcinoma	Parotid	NA	NB	NA	NB	NB	NB	NB
Mucoepidermoid carcinoma	Parotid	NA	NB	NA	NB	NB	NB	B
Mucoepidermoid carcinoma	Parotid	NA	B	NA	NB	NB	NB	NB
Mucoepidermoid carcinoma	Parotid	NA	B	NA	NB	NB	B	B
Mucoepidermoid carcinoma	Parotid	NA	NB	NA	NB	NB	NB	NB
Mucoepidermoid carcinoma	Parotid	NA	B	NA	NB	NB	NB	NB
Mucoepidermoid carcinoma	Parotid	NA	B	NA	NB	NB	NB	auSNB
Mucoepidermoid carcinoma	Parotid	NA	NB	NA	auSNB	NB	NB	NB
Mucoepidermoid carcinoma	Parotid	NA	NB	NA	NB	NB	NB	NB
Mucoepidermoid carcinoma	Parotid	NA	NB	NA	auSNB	NB	NB	auSNB
Adenoid cystic carcinoma	Parotid	NA	NB	NA	auSNB	NB	NB	NB
Adenoid cystic carcinoma	Parotid	NA	NB	NA	NB	NB	NB	NB
Adenoid cystic carcinoma	Parotid	NA	NB	NA	NB	NB	NB	NB
Adenoid cystic carcinoma	Minor salivary gland	NA	NB	NA	NB	NB	NB	NB
Adenoid cystic carcinoma	Submandibular gland	NA	NB	NA	NB	NB	NB	NB
Adenoid cystic carcinoma	Parotid	NA	NB	NA	NB	NB	NB	NB
Adenoid cystic carcinoma	Submandibular gland	NA	NB	NA	NB	NB	NB	NB
Acinic cell carcinoma	Parotid	NA	NB	NA	NB	NB	NB	NB
Acinic cell carcinoma	Parotid	NA	NB	NA	NB	NB	B	NB
Acinic cell carcinoma	Parotid	NA	NB	NA	NB	NB	NB	NB
Acinic cell carcinoma	Parotid	NA	NB	NA	NB	NB	B	NB
Acinic cell carcinoma	Parotid	NA	NB	NA	NB	NB	NB	auSNB
Carcinoma ex-pleomorphic adenoma	Parotid	NA	NB	NA	NB	NB	NB	miniB
Carcinoma ex-pleomorphic adenoma	Parotid	NA	NB	NA	NB	NB	NB	NB
Carcinoma ex-pleomorphic adenoma	Parotid	NA	NB	NA	NB	NB	NB	auSNB
Carcinoma ex-pleomorphic adenoma	Parotid	NA	NB	NA	auSNB	NB	NB	auSNB
Myoepithelial carcinoma	Parotid	NA	NB	NA	NB	NB	NB	NB
Myoepithelial carcinoma	Minor salivary gland	NA	NB	NA	auSNB	NB	NB	auSNB
Squamous carcinoma	Parotid	NA	NB	NA	auSNB	NB	NB	NB
Squamous carcinoma	Parotid	NA	NB	NA	auSNB	NB	NB	B
Adenocarcinoma, NOS	Minor salivary gland	NA	mini B	NA	NB	NB	NB	NB
Epithelial-myoepithelial carcinoma	Parotid	NA	NB	NA	auSNB	NB	NB	NB
Neuroendocrine carcinoma	Parotid	NA	NB	NA	NB	NB	NB	NB
Salivary ducts carcinoma	Parotid	A	NB	NA	NB	NB	NB	NB

Of these four patients, 3 are women, all over 50 years of age at the time of diagnosis, while the male subject was 27. The tumors were all located in the parotid gland. Three were located in levels I and II according to ESGS [13], and one was located in levels II and IV according to ESGS [13].

Among the patients, three initially presented with painless masses, while one experienced a painful swelling. The median time from the onset of symptoms to diagnosis was 14 months, with a range spanning from 6 weeks to 20 years. Notably, none of these patients received an initial diagnosis of secretory carcinoma, although three were initially diagnosed before 2017.

In both mucoepidermoid carcinomas, the rearrangement of MALM2 was found in addition to that of NTRK3, while in only one of the two also of EWRS1. Two of these patients were in stage cT1N0M0 at the time of diagnosis (1 mucoepidermoid and 1 acinic cell carcinoma), one in stage cT4aN0M0 (mucoepidermoid carcinoma), and one in stage cT2N0M0 (acinic cell carcinoma). All these patients underwent surgery; only one underwent adjuvant radiotherapy treatment (cT4aN0M0).

The primary treatment for all patients involved surgical resection of the parotid gland, with two undergoing resection of all levels, including the deep ones. In contrast, the other two underwent resection of only the superficial levels. In one case, the surgical procedure included an ipsilateral selective neck dissection encompassing levels IIa, IIb, III, and IV, but no metastatic lymph nodes were detected. All patients achieved clear resection margins. Notably, only one patient experienced local recurrence twice, with the first occurring 30 months after the initial diagnosis. The patients’ grading was G1 in every case except for those who experienced recurrences, whose grading was G2.

The average duration of follow-up for these patients is 78.5 months, spanning from 49 to 112 months.

### Survival Outcomes

**Fig. 1:**
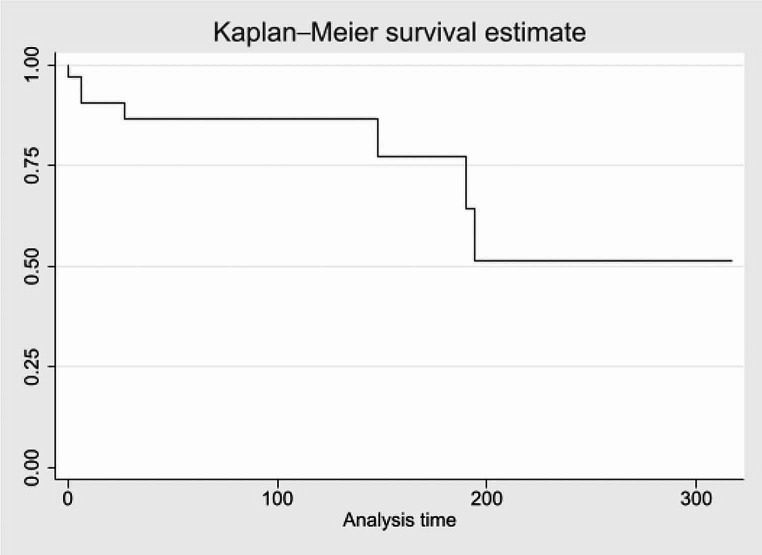
OS, time expressed in months

The study observed that 90% of the sample survived beyond 6 weeks. At 122 weeks, the survival rate was 87%. By 160 weeks, the survival rate decreased to 77%; at 194 weeks, 51% of the sample remained alive. OS of all the patients included is summarized in Fig. 1.

**Fig. 2:**
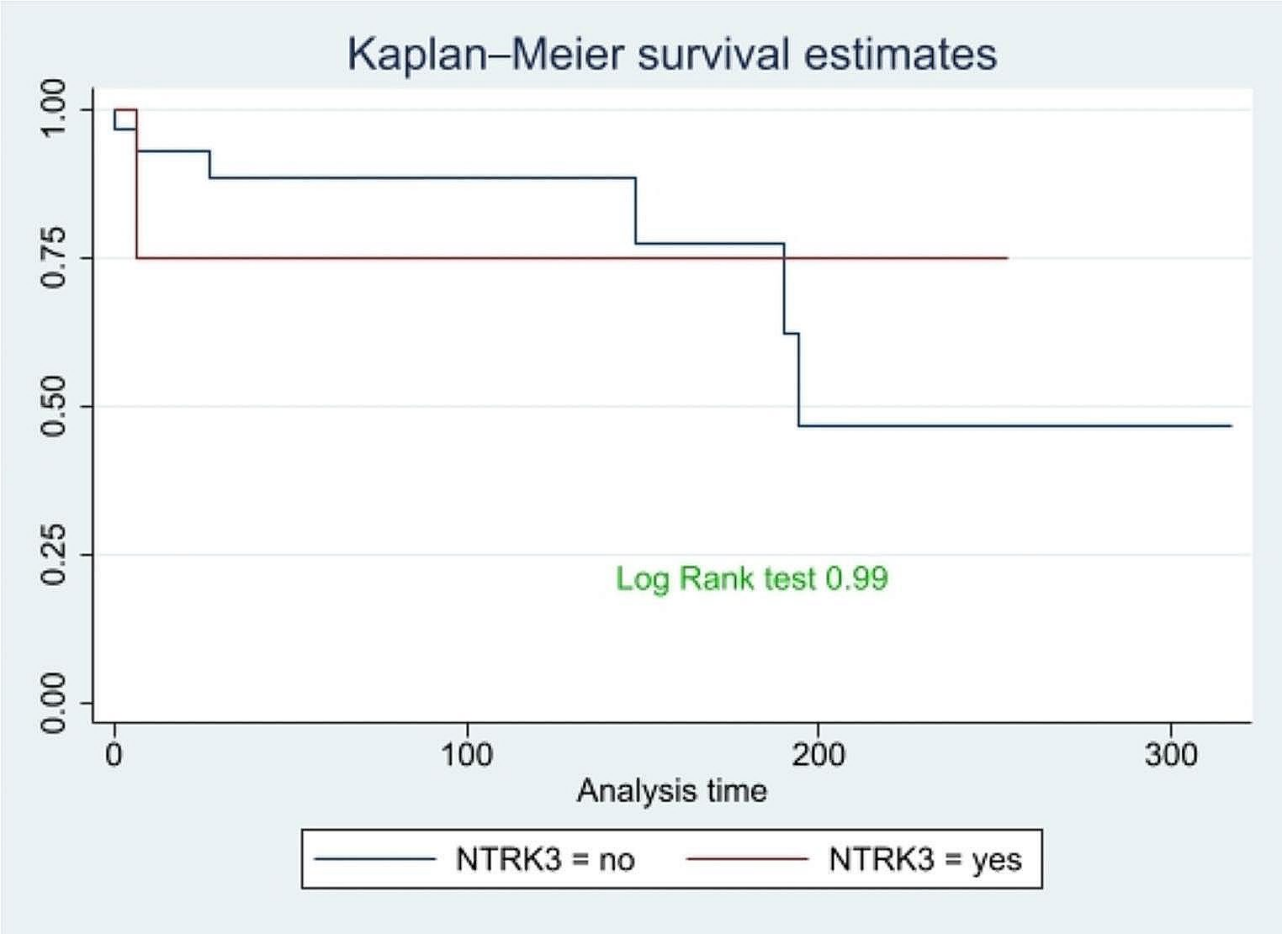
Comparison of OS between NTRK3 rearrangement patients and the others, time expressed in months

Figure 2 shows OS of patients with the NTRK3 gene mutation compared to others. No significant inferences can be drawn from this graph, as the p-value from the log-rank test is not significant (p-value = 0.99).

Regarding PFS, among all the patients followed for up to 316 months, 7 experienced relapses. The data indicate that 96% of the sample remains relapse-free beyond 21 weeks, 80% beyond 54 weeks, 72% beyond 122 weeks, and 63% beyond 129 weeks.

**Fig. 3:**
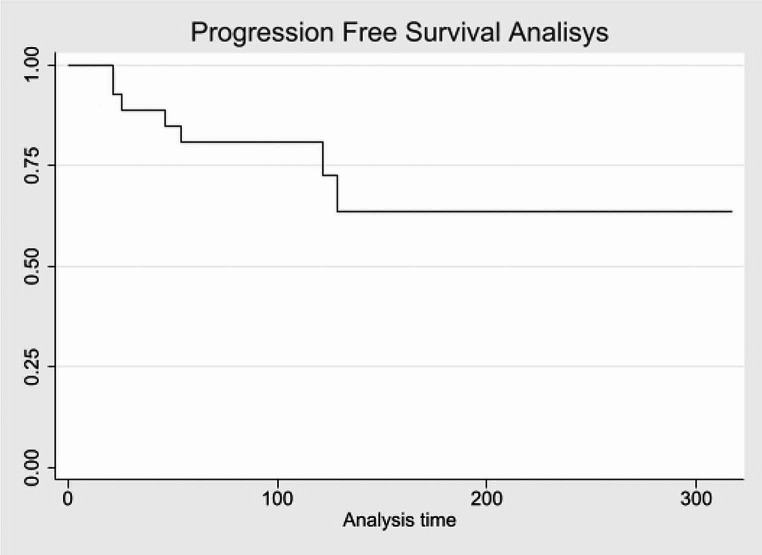
PFS, time expressed in months

These data are illustrated in Fig. 3.

**Fig. 4:**
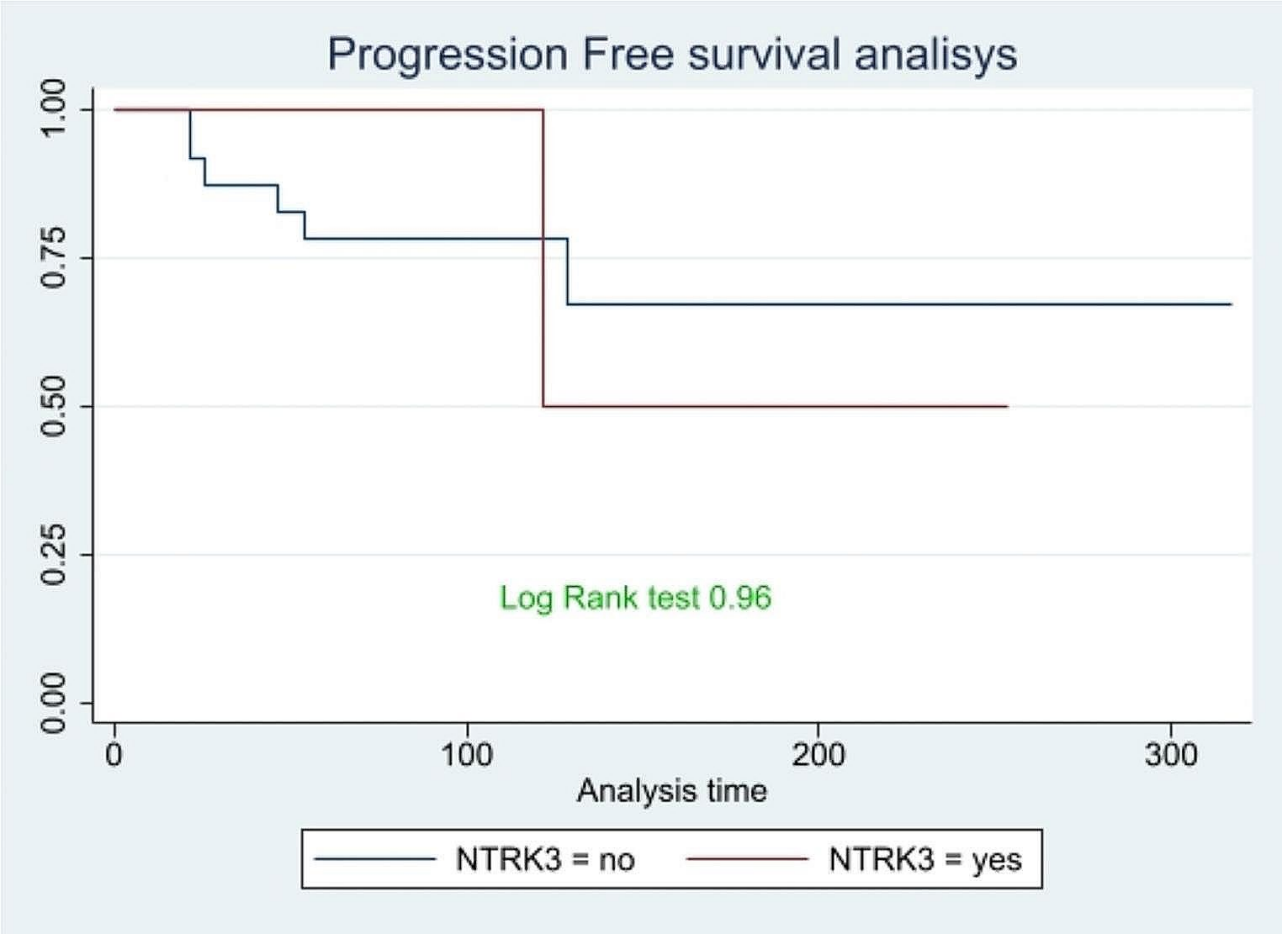
Comparison of PFS between NTRK3 rearrangement patients and the others, time expressed in months

Figure 4 shows PFS of patients with the NTRK3 gene mutation compared to others. No significant inferences can be drawn from this graph, as the p-value from the log-rank test is not significant (p-value = 0.96).

Regarding NTRK3-mutated group, as summarized in Table 3, patient 1, diagnosed with acinic cell carcinoma, had two recurrences but was disease-free at the last checkup in July 2023. Her OS was 48 months, and PFS to the first recurrence was 30 months. Patient 2 was diagnosed with mucoepidermoid carcinoma and passed away in July 2021 due to causes unrelated to cancer. Her OS was 51 months, and her PFS was also 51 months. Patient 3 was diagnosed with mucoepidermoid carcinoma and was disease-free at the last checkup in July 2023. OS and PFS are both 111 months. Patient 4 was diagnosed with acinic cell carcinoma and was disease-free at the last checkup in July 2023. Her OS and PFS are 102 months.

The collective OS for patients with NTRK3 rearrangements is 78.5 months, while the overall PFS is 73.75 months. In the subset of two mucoepidermoid carcinomas, the OS spans 75.5 months, and the PFS extends to 81 months. Conversely, the OS mirrors the PFS at 81.5 months for the two acinic cell carcinomas. This data is resumed in Table 3.

**Table 3:** Survival of NTRK3-mutated patients

Patient	Gender and age at diagnosis	Histology	Clinical staging	Treatment	Overall Survival (months)	Disease-free survival (months)
1	F, 72	Mucoepidermoid carcinoma	T2N0M0	S	49	30
2	F, 87	Acinic cell carcinoma	T4aN0M0	S + RT	51	51
3	M, 27	Acinic cell carcinoma	T1N0M0	S	112	112
4	F, 54	Mucoepidermoid carcinoma	T1N0M0	S	102	102

Female (F), Male (M)

Surgery (S), Radiotherapy (RT)

The OS and PFS of patients with mucoepidermoid carcinoma without NTRK3 rearrangement are 70.22 months and 67.33 months, respectively. The OS and PFS of patients with acinic cells without NTRK3 rearrangement are 113 months and 50 months, respectively.

## Discussion

Secretory carcinoma is a recently identified carcinoma distinguished by its molecular hallmark, the ETV6-NTRK3 fusion, which is known to drive oncogenic processes. While extensive research has been conducted on the histopathological characteristics of secretory carcinoma in existing literature, there needs to be more investigations on its clinical behavior.

This report delves into the clinical profile of secretory carcinoma in a cohort of four patients, all of whom tested positive for the NTRK3 gene rearrangement. Notably, the male-to-female ratio within this cohort was 1:3. This contrasts with a broader literature review encompassing 279 cases, where the male-to-female ratio stood at 1.5:1 [14]. Furthermore, according to this review, secretory carcinoma tends to present at a comparatively younger age, with the average age of diagnosis being 45 years. Furthermore, it is common for this condition to be diagnosed in the pediatric population [14]. It is worth acknowledging that the observed ratio variation within this case series is influenced by its small sample size.

Our observations included cases categorized as T1, T2, and T4a, which aligns with findings reported in other case series in the existing literature [15]. This data can be interpreted in two ways: on one hand, the behavior of secretory carcinomas appears to be less aggressive than their mimickers, which might lead to an expectation of later diagnosis and a more advanced stage, even in the absence of significant symptoms. On the other hand, their slow growth could result in an earlier-stage diagnosis. However, further scientific evidence and a prospective collection of a larger sample size are necessary [15].

In most retrospective studies, secretory carcinoma was formerly categorized as acinic cell carcinoma. Even within our patient cohort, two cases had received such classification before immunohistochemical examination. Acinic cell carcinoma can be morphologically distinguished through its structural and cytological diversity and large serous acinar cells containing zymogen granules, a characteristic absent in secretory carcinoma [16].

Per existing literature, our case series exclusively featured tumors within the parotid gland. In fact in a comprehensive review encompassing 279 cases, the predominant tumor locations were the parotid gland (68%), followed by the minor salivary glands of the buccal mucosa (9%), submandibular gland (8%), minor salivary glands of the lower lip (5%), minor salivary glands of the upper lip (4%), and minor salivary glands of the hard palate (4%). Isolated cases were reported in the minor salivary glands of the soft palate, with a singular case noted at the base of the tongue (collectively, 2%) [14].

Interestingly, secretory carcinoma appears more commonly found outside the parotid gland than acinic cell carcinoma. Consequently, when patients are diagnosed with acinic cell carcinoma in the minor salivary glands, it should raise suspicions of the possibility of secretory carcinoma [17].

In this report, only one patient encountered two instances of local recurrence. The first recurrence was on the primitive tumor site and was treated by resectioning the remaining parotid levels and a selective neck dissection, including levels IIa, IIb, III, and IV. The second recurrence was on the neck and was treated through radiotherapy. Presently, the patient has achieved a disease-free status.

Distant metastasis was not observed in our patient group. Nevertheless, it is essential to exercise caution when interpreting these findings. This caution arises from the fact that the mean follow-up duration in this study is just 78.5 months. Considering the typically low-grade behavior of secretory carcinoma, local recurrences might emerge even after a more extended follow-up period. Reports exist in the literature of patients diagnosed with tumors that underwent high-grade transformation, with some sadly succumbing to disseminated disease within a relatively short span [18, 19].

In the present study, the disease’s progression demonstrates a favorable course concerning OS and PFS and appears similar to acinic cell carcinoma. Indeed, in a study utilizing data from the National Cancer Database, researchers identified 1,353 cases of acinic cell carcinoma within the head and neck region. The findings revealed a five-year survival rate of 83.3%, and a more focused measure, disease-specific survival rate, stood at 91.4%. Notably, factors linked to diminished survival encompassed higher-grade tumors (*p* < 0.0001), individuals aged 30 years or older (*p* = 0.0055), and the presence of metastatic disease (*p* < 0.0001) [20].

Nonetheless, it is crucial to consider that the favorable prognosis observed in our study may be attributed to the predominance of early-stage cases and the generally low-grade nature of the disease among most of our patients.

While acinic cell tumors and secretory carcinomas of the salivary glands may share similar clinical behaviors, differentiating secretory carcinomas from other histological types is of utmost significance, mainly when metastases are present. Identifying the NTRK-ETV6 fusion gene has unveiled a targetable opportunity, and an ongoing clinical trial is dedicated to this pursuit.

In this clinical trial, currently in phase 2, an examination was conducted involving patients affected by diverse advanced solid cancers characterized by NTRK3 fusion. The findings indicate that entrectinib (a potent inhibitor of TRK A, B, and C, which has been shown to have anti-tumor activity against NTRK gene fusion) exhibits efficacy across multiple tumor categories, demonstrating both systemic anti-tumor effects and effectiveness in managing metastases within the central nervous system. Notably, disease control was sustained, with a median progression-free survival of 11 months and a median response duration of 10 months [21].

The matter remains far from conclusive regarding adjuvant radiotherapy in acinic cell carcinomas. A recent study involving 187 patients observed that individuals with cN0 acinic cell carcinoma without high-grade transformation, who underwent both surgery and radiotherapy, did not exhibit enhanced PFS compared to those who underwent surgery alone. Regarding neck dissection, the same study attributes correlated with hidden nodal invasion encompassed the T3-T4 tumor stage and intermediate/high histological grading. After propensity score matching, a proactive neck dissection demonstrated a tendency toward enhanced PFS [22].

In contrast to acinic cell carcinoma, secretory cell carcinoma demonstrates a slightly increased propensity for lymph node metastasis, particularly in cases with high-grade pathology. This suggests a potential need for neck dissection and adjuvant radiotherapy in intermediate/high-grade cases and advanced T stages [19]. Regarding distant metastases, similar to acinic cell carcinomas, some secretory carcinomas exhibit an aggressive course, and it remains challenging to consistently identify histological or staging factors that reliably predict an adverse outcome. However, it is notable that they often present in high-grade tumors, irrespective of the T stage [12].

In our case series, the behavior of secretory carcinomas is, therefore, quite favorable, with good OS and PFS. The patient who received the diagnosis at a more advanced stage had had symptoms of the disease for some time; this suggests that with a low grading, as in this case, the disease tends to grow slowly. However, as regards the case which developed two relapses, it should be underlined that it was an intermediate grading that had not carried out treatment on the neck or adjuvant therapy. This may suggest that the attitude of non-low-grade tumors should be more aggressive.

While acknowledging the intrinsic limitations inherent in our case series, characterized by relatively low statistical power, our findings align with existing literature. The differentiation of secretory carcinomas of the salivary glands assumes paramount importance, not only in distinguishing them from acinic cell carcinomas, with which they are frequently confused, but also from other histological subtypes. This differentiation holds significance in shaping therapeutic strategies, where surgery remains the gold standard, and in guiding decisions related to neck management and adjuvant treatments. Neck dissection may be warranted even in cN0 cases with low T stage but intermediate and high grading. Regarding radiotherapy, a potential survival benefit emerges primarily in cases of high-grade tumors. In contrast, identifying other risk factors indicative of adjuvant radiotherapy benefits remains an area for further investigation.

It is imperative to collect a more extensive dataset to formulate an effective strategy for managing secretory carcinomas. This would provide substantial insight regarding the need for neck dissection and postoperative adjuvant therapy. Conversely, there are already promising findings about the efficacy of targeted therapies for metastatic patients.

## Conclusions

The quest to detect the ETV6-NTRK3 rearrangement, a hallmark of secretory carcinomas, underscores its paramount significance. The true prevalence of secretory carcinoma is likely underestimated, particularly considering recent histological reclassifications of salivary gland tumors, thus emphasizing the need for routine molecular profiling. Given the absence of distinct morphological hallmarks characterizing this neoplasm, it is advisable to employ FISH analysis utilizing the NTRK3 probe when morphological indicators suggest a potential differential diagnosis.
